# Potential role of astrocyte angiotensin converting enzyme 2 in the neural transmission of COVID-19 and a neuroinflammatory state induced by smoking and vaping

**DOI:** 10.1186/s12987-022-00339-7

**Published:** 2022-06-07

**Authors:** Yong Zhang, Sabrina Rahman Archie, Yashwardhan Ghanwatkar, Sejal Sharma, Saeideh Nozohouri, Elizabeth Burks, Alexander Mdzinarishvili, Zijuan Liu, Thomas J. Abbruscato

**Affiliations:** 1 Department of Pharmaceutical Sciences, Texas Tech University Health Sciences Center School of Pharmacy, Amarillo, TX USA; 2 Imaging Core at Office of Sciences, Texas Tech University Health Sciences Center School of Pharmacy, Amarillo, TX USA

**Keywords:** COVID-19, SARS-CoV-2, ACE2, Astrocyte, Inflammation, Neurogenesis, Smoking

## Abstract

**Background:**

Knowledge of the entry receptors responsible for SARS-CoV-2 is key to understand the neural transmission and pathogenesis of COVID-19 characterized by a neuroinflammatory scenario. Understanding the brain distribution of angiotensin converting enzyme 2 (ACE2), the primary entry receptor for SARS-CoV-2, remains mixed. Smoking has been shown as a risk factor for COVID-19 severity and it is not clear how smoking exacerbates the neural pathogenesis in smokers.

**Methods:**

Immunohistochemistry, real-time PCR and western blot assays were used to systemically examine the spatial-, cell type- and isoform-specific expression of ACE2 in mouse brain and primary cultured brain cells. Experimental smoking exposure was conducted to evaluate the effect of smoking on brain expression.

**Results:**

We observed ubiquitous expression of ACE2 but uneven brain distribution, with high expression in the cerebral microvasculature, medulla oblongata, hypothalamus, subventricular zones, and meninges around medulla oblongata and hypothalamus. Co-staining with cell type-specific markers demonstrates ACE2 is primarily expressed in astrocytes around the microvasculature, medulla oblongata, hypothalamus, ventricular and subventricular zones of cerebral ventricles, and subependymal zones in rhinoceles and rostral migratory streams, radial glial cells in the lateral ventricular zones, tanycytes in the third ventricle, epithelial cells and stroma in the cerebral choroid plexus, as well as cerebral pericytes, but rarely detected in neurons and cerebral endothelial cells. ACE2 expression in astrocytes is further confirmed in primary cultured cells. Furthermore, isoform-specific analysis shows astrocyte ACE2 has the peptidase domain responsible for SARS-CoV-2 entry, indicating astrocytes are indeed vulnerable to SARS-CoV-2 infection. Finally, our data show experimental tobacco smoking and electronic nicotine vaping exposure increase proinflammatory and/or immunomodulatory cytokine IL-1a, IL-6 and IL-5 without significantly affecting ACE2 expression in the brain, suggesting smoking may pre-condition a neuroinflammatory state in the brain.

**Conclusions:**

The present study demonstrates a spatial- and cell type-specific expression of ACE2 in the brain, which might help to understand the acute and lasting post-infection neuropsychological manifestations in COVID-19 patients. Our data highlights a potential role of astrocyte ACE2 in the neural transmission and pathogenesis of COVID-19. This also suggests a pre-conditioned neuroinflammatory and immunocompromised scenario might attribute to exacerbated COVID-19 severity in the smokers.

## Introduction

The coronavirus disease 2019 (COVID-19) pandemic, caused by the new severe acute respiratory syndrome coronavirus 2 (SARS-CoV-2), has become a global health emergency. The most common manifestation in COVID-19 patients are respiratory symptoms and pulmonary infection; however, a growing body of evidence shows extrapulmonary symptoms are also common in COVID-19 patients [1, 2]. Approximately, 30–80% of patients experience various neuropsychological symptoms such as smell and taste loss, dizziness, neuralgia, seizures, encephalitis, cognitive disorder, memory impairment, stroke, or rare Guillain–Barre syndrome [1–6]. Over one third of patients recovered from the disease have lasting post-infection neuropsychological manifestations collectively referred to as post-acute sequelae of SARS-CoV-2 infection (PASC) [7, 8]. Several studies have reported the presence of SARS-CoV-2 in brain [9–11] or cerebrospinal fluid (CSF) [12], suggesting a possible brain invasion of SARS-CoV-2. It is worthy to note, the detection of positive SARS-CoV-2 in CNS is much lower compared to that in the pulmonary system of patients with severe cardio-pulmonary manifestations, raising a caution of potential contamination during sample collecting and processing. Whereas, positive SARS-CoV-2 in CSF of certain patients with mild symptoms and/or lacking significant cardio-pulmonary manifestations implies a possible neural invasion in these patients [13–19]. Postmortem examinations of COVID-19 patients reveal a neuroinflammatory scenario in the central neural system (CNS) characterized by activation of glial cells and infiltration of inflammatory cells [9, 20, 21]. Glial cells including astrocytes and microglia have key functions in maintaining CNS homeostasis and responding to physical, infectious, and neurodegenerative disease-related insults via producing and releasing pro- and/or anti-inflammatory cytokines and chemokines, antioxidants, free radicals, and neurotrophic factors [22, 23]. Given that the large number of functions performed by glial cells in the neuroinflammatory response, it could be expected that activation of astrocytes has a major impact on the CNS during SARS-CoV-2 infection [23, 24].

Angiotensin converting enzyme 2 (ACE2) functions as the primary receptor for SARS-Cov-2 entry [25]. ACE2 is a transmembrane protein, in which the extracellular peptidase domain (PD) has high affinity to the receptor-binding domain (RBD) of the spike (S) protein of SARS-CoV-2 [26]. After binding to the PD, the S protein is primed by surface proteinases such as TMPRSS2, TMPRSS11A/B, cathepsin B/L and FURIN to facilitate SARS-CoV-2 entry into host cells followed by deregulation of membrane ACE2 [27, 28]. In addition to membrane ACE2, intracellular ACE2 and soluble ACE2 have also been identified, although they are incapable of facilitating SARS-CoV-2 entry. ACE2 is an essential counteractor in the multifunctional renin-angiotensin system (RAS) by modulating angiotensin (Ang) peptide balance. The primary function of ACE2 in the RAS is to convert the Ang I and Ang II to Ang 1–9 and Ang 1–7, respectively [29]. Overactive Ang II signaling promotes inflammation and injuries in multiple tissues and organs. ACE2 ameliorates inflammatory injuries via controlling the ratio of pro-inflammatory Ang II to anti-inflammatory Ang 1–7. Phase II clinical trials have shown administration of ACE2 reduces inflammation that is associated with a shift of Ang peptide balance away from Ang II toward Ang 1–7 [30, 31]. It could be expected that deregulation of ACE2 by SARS-CoV-2 or S protein shed from SARS-CoV-2 might lead to overactive Ang II signaling. Consistent with this, a recent study shows Ang II levels in COVID-19 patients are elevated and linearly correlated to the viral load and lung injuries, suggesting an imbalance of Ang peptides might, in part, attribute to the development of inflammatory injuries in COVID-19 patients[29, 32].

In view of the critical role of ACE2 in mediating SARS-CoV-2 entry and protecting inflammatory injuries, knowledge of its brain distribution is informative to understand the neural transmission and pathogenesis of COVID-19. However, understanding its brain distribution remains mixed. A number of research papers and comprehensive reviews have been published on the ACE2 expression in the brain. A recent study performed on the rat brain reports that ACE2 expression in brain capillaries, hindbrain pontine nucleus, pre-Bötzinger complex, nucleus of tractus solitarius, and in neurons, astrocytes, pericytes and endothelial cells. The authors conclude that ACE2-expressing neurons may play a possibly important role in the neural manifestations in COVID-19 patients [33]. A computational analysis of human and mouse brain databases shows ACE2 is expressed in the choroid plexus, paraventricular nuclei of the thalamus and olfactory bulbs, as well as in neurons, astrocytes, oligodendrocytes, pericytes and endothelial cells. ACE2 expression has also been reported in the cultured astrocytes, microglial cells, pericytes and endothelial cells [34]. Whereas other studies show neurons, microglial cells and endothelial cells have rare or undetectable ACE2 expression [11, 35–37]. It remains to be determined which brain region(s) and cell type(s) express relatively high ACE2 levels and therefore might be vulnerable to SARS-CoV-2. In addition, it is also not clear by which route(s), such as blood brain barrier (BBB), blood-CSF barrier (BCSFB) or retrograde olfactory migration, SARS-CoV-2 might be more likely to invade into the CNS, particularly in patients without significant BBB impairment. Furthermore, smoking has been reported as a risk factor for COVID-19 severity in current smokers [38, 39]. Previous studies have shown smoking upregulates pulmonary ACE2, which has been considered to contribute to the infection susceptibility, disease severity and treatment outcome in COVID-19 patients [40–42]. Whereas it remains unclear if smoking exacerbates CNS pathogenesis in a similar or alternative manner. It is also not clear if electronic nicotine vapor (commonly known as electronic cigarette) would harm the lungs or CNS in a similar manner to tobacco cigarettes.

In the current study, we systemically examined the brain distribution of ACE2 and explored the impact of smoking on the mouse brain distribution of ACE2 and the induction of a neuroinflammatory state. Our data elucidate a spatial- and cell type-specific distribution of ACE2 and a pre-conditioned neuroinflammatory state by smoking exposure in the CNS, which might help understand the neural transmission and pathogenesis of COVID-19 in smokers and nonsmokers.

## Methods

### Frozen tissue section preparation

CD-1 and C57/BL/6 J mice at 8–10 weeks of age were obtained from the Charles River Laboratories. After euthanasia, the whole brains were carefully dissected and sectioned at 20 µm of thickness with a Leica cryostat (Leica Microsystem), then the tissue sections were mounted on glass slides for latter assays. All animal studies were approved by the Texas Tech University Health Sciences Center Institutional Animal Care and Use Committees (IACUC).

### Primary cell culture

Primary astrocytes, neurons and brain microvascular endothelial cells (BMEC) were isolated from CD-1 mice. BMECs were isolated and cultured following previous protocol [43]. Briefly, adult brains were dissected and cut into small pieces, then incubated with 5 ml of papain and 250 µl of 2000U DNase I (Worthington Biochemical Corp) at 37 °C for 70 min. The digested brain tissues were passed through 19-gauge and 21-gauge needles, then mixed with equal volume of 22% bovine serum albumin (BSA) solution to centrifuge at 1360 g for 10 min. The tissue pellet was resuspended in complete growth medium (Nutrient Mixture F12 Ham supplemented with 10% fetal bovine serum (FBS), 1% penicillin/streptomycin, endothelial cell growth supplement, ascorbate, L -glutamine and heparin). Puromycin were used for purity selection for 2.5 days. The complete growth medium was refreshed every 2 days until reaching confluence. Neurons were isolated and cultured as previously described [44]. In brief, mouse cortices were isolated from E16 or E17 embryos and meninges were removed in the Hank's balanced salt solution (HBSS, without Ca^2+^ and Mg^2+^) supplemented with 250 µg/ml gentamycin. The cortices were digested by 0.25% trypsin for 15 min at 37 °C followed by neutralization with the complete growth medium (neurobasal medium, 0.5 mM glutamine, 25 µg/ml gentamicin and 2% B27 supplement [Thermo Fisher]) plus 10% FBS. After centrifuge, cells were resuspended and seeded (1.2 × 10^4^ per cm^2^ surface area) onto 6-well plates coated with poly-D-lysine (Sigma-Aldrich). The complete growth medium was totally replaced after overnight and then half of the medium was refreshed every 2 days until day 7–9. Astrocytes were isolated and cultured following the previous protocol with a little modification [45]. After isolating the newborn brains, cortices were removed and placed in HBSS (without Ca^2+^ and Mg^2+^) supplemented with gentamycin at 250 µg/ml. Then cortices were digested with 0.25% trypsin for 15 min at 37 °C followed by neutralizing with the complete growth medium (DMEM containing 10% FBS and 1% penicillin/streptomycin) (Thermo Fisher). The cells were then seeded into cell culture flasks. Growth medium was refreshed every 3 days until reaching confluency. All cells were maintained at 37 °C supplementary with 5% CO2 environment.

### Immunohistochemistry (IHC)

IHC staining was performed as previously described [46]. The frozen brain sections were fixed with 4% paraformaldehyde (Thermo Fisher) for 15 min, then permeabilized with 0.1% Trion X-100 for 10 min. After washing with the phosphate-buffered saline (PBS), the sections were blocked with 6% horse serum in 1% BSA for 1 h, then incubated overnight with the indicated antibodies against ACE2 and Nestin (1:100, 1: 100, Cat# AF933 and NB100-1604, R&D system), CD31 (1:200, Cat# 550,274, BD Biosciences), PDGRFβ, NeuN and GFAP (1:200, 1:500, 1:500, Cat# 3169, 12389 and 24307, Cell Signaling Technology), and Claudin-3 (1:100, Cat# 34–1700, Thermo Fisher). Alexa Fluorescent secondary antibodies (Thermo Fisher) were used at 1:200 dilutions for 1 h. After counterstaining with 4′,6-diamidino-2-phenylindole (DAPI) and washing with PBS, the sections were mounted with Permount (Thermo Fisher). The whole sections were scanned with a Leica Stellaris SP8 Falcon microscope (Leica Microsystem) and the other images were captured with the same microscope or a Nikon A1RMP confocal microscope (Nikon Instrument). ACE2 signal intensity was analyzed with the NIS-Elements AR analysis software (Nikon Instrument). Sixty cells of astrocyte and neuron were randomly chosen from the comparable regions out of 6 sections to evaluate the relative expression levels of ACE2 in each cell type. To minimize subjective bias, all images for ACE2 expression analysis were captured under the same microscopic parameter (laser power, pinhole size, exposure time) setting.

### RNA isolation and real-time polymerase chain reaction (PCR)

RNA was extracted from primary astrocytes, neurons and BMECs by using Trizol reagent (Thermo Fisher) according to the user manual. Total RNA (2 μg) from each sample was reverse transcribed by using iScript reverse transcription supermix (Bio-Rad Laboratories). Real-time PCR was performed on a C1000 Touch Thermal Cycler (Bio-Rad Laboratories) using iTaq Universal SYBR^®^ Green Supermix (Bio-Rad Laboratories) as previous described [47]. ACE2 primer 1: Forward 5ʹ-TCCATTGGTCTTCTGCCATCC-3ʹ, Reverse 5ʹ-AACGATCTCCCGCTTCATCTC-3ʹ. ACE2 primer 2: Forward 5ʹ-TTTGTTTCTGTTGGCCTTCC-3ʹ, Reverse 5ʹ-CATTGGCTCCGTTTCTTAGC-3ʹ. β-actin was used for normalization. The PCR condition was started as 1 cycle of 2 min at 50 °C and 10 min at 95 °C followed by 40 cycles of 15 s at 95 °C and 1 min at 60 °C.

### Western blot

The whole cell lysate was extract from primary astrocytes, neurons and BMECs for immunoblotting as described previously [48]. In brief, the cells were lysed with radioimmunoprecipitation assay buffer (RIPA) buffer containing proteinase inhibitor and the protein concentration was determined by BCA assays (Thermo Fisher). Equal amount of protein from each sample was separated in 10% PAGE gel, and then transferred to nylon membrane blot. After blockage with 5% non-fat milk, the blot was incubated with primary antibodies against ACE2 (Cat# AF933, R&D system) and β-actin (Millipore Sigma) in 1% BSA for overnight. After incubate with the secondary antibody for 1 h, the blot was developed with X-ray films. β-actin was used as a loading control. Image J software (National Institutes of Health [NIH]) was used to quantitate the band intensity.

### Experimental smoking exposure

C57BL/6 male mice were exposed (via direct inhalation) to 3R4F standardized research tobacco cigarettes (9.4 mg tar and 0.726 mg nicotine/cigarette equivalent to full flavor commercial products, University of Kentucky) or Juul electronic nicotine vapor (50 mg/ml nicotine) mixed with oxygenated air or oxygenated air alone, at 6 cycles/day exposure rate for 14 days. The tobacco smoke (TS) or electronic nicotine vapor exposure was conducted between 9 am to 2 pm every day. A modified CIR (Canadian Intense Regimen) standard smoking protocol (27.5 ml puff depth volume, 2 s puff duration, 2 puffs per 60 s, 32 puffs/cycle) to study TS exposure and a modified CORESTA (Cooperation Centre for Scientific Research Relative to Tobacco) standard smoking protocol to study Juul electronic nicotine vapor exposure (27.5 ml puff depth volume, 3 s puff duration, 2 puffs per 60 s, 32 puffs/cycle) were followed in the laboratory. TS and Juul vapor were generated using a Single Cigarette Smoking Machines (SCSM, CH Technologies Inc) following a previously published method [49]. These methods were followed to mimic the smoking behavior of a human current tobacco smoker. At the end of the experiments, mouse brain tissues were homogenized in RIPA buffer supplemented with proteinase inhibitors. The tissue lysate was used for western blot assays and inflammatory cytokines assays with the Mouse High Sensitivity T-Cell 18-Plex Discovery Assay® Array (MDHSTC18) (Eve Technologies, Canada). The data were normalized by protein concentrations.

### Statistical analysis

One-way ANOVA, two-way ANOVA and t-test were used to determine the statistical significance among or between the specific groups. Normality tests were performed firstly. Non-parametric tests were employed for the non-normality data. Dunnett and Šídák tests were used as the post-hoc tests for multiple comparisons of normally distributed data, and Dunn test were used for multiple comparisons of non-normally distributed data. All statistical analyses and graphs were generated by the Prism (v9.0) software (GraphPad Software). P value < 0.05 was considered as statistically significant.

## Results

### Expression of ACE2 in cerebral microvascular pericytes

**Fig. 1:**
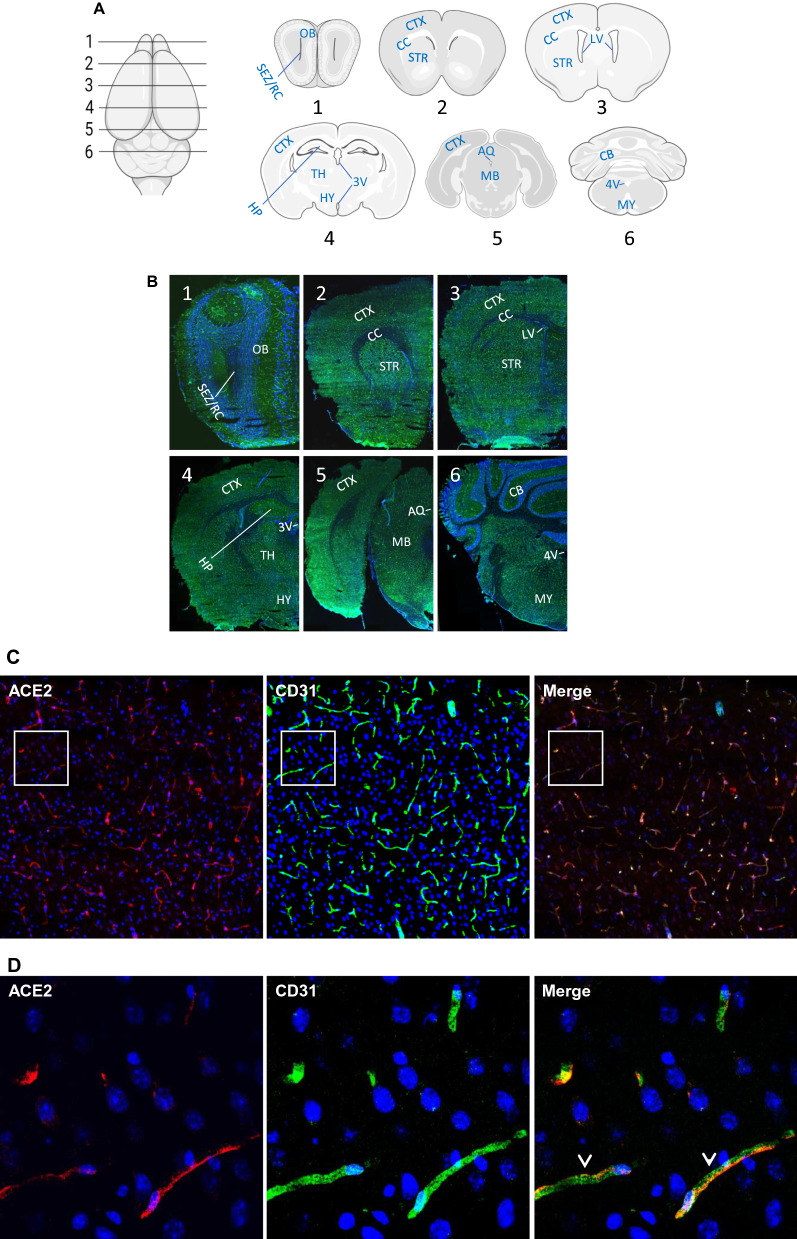

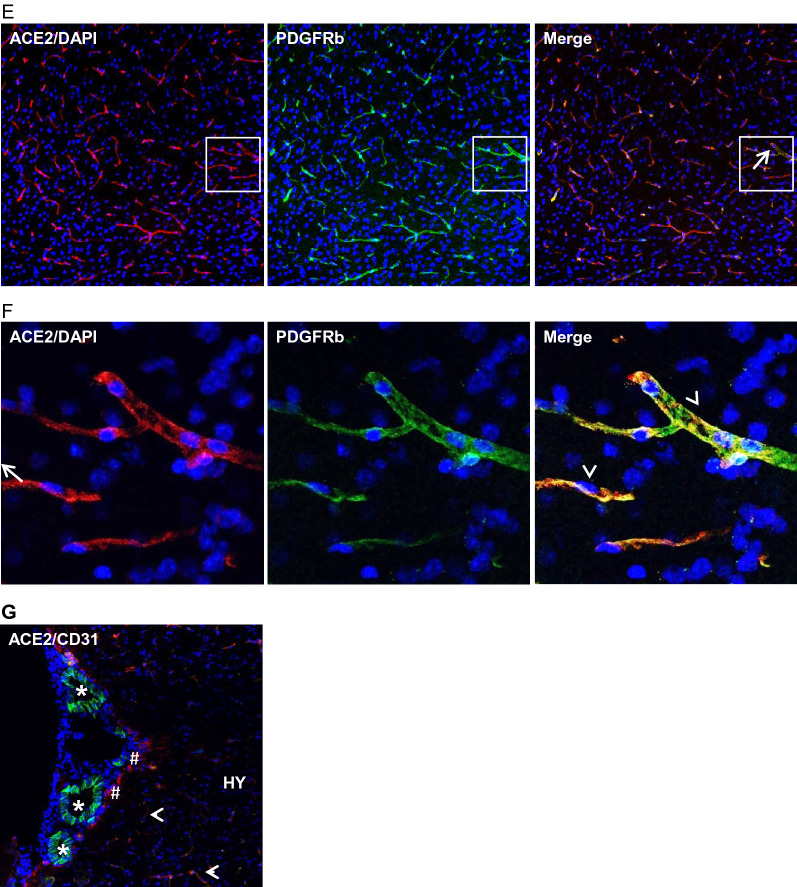
Ubiquitous expression of ACE2 in cerebral microvascular pericytes. **A** Schematic elucidation of sectioning plates and main brain regions of a mouse brain. OB: olfactory bulb, SEZ/RC: subependymal zone (SEZ) in the rhinocele, *CTX*: cerebral cortex, *CC*: corpus callosum, *STR*: striatum, *LV*: lateral ventricle, *HP*: hippocampus, *TH*: thalamus, 3 V: third ventricle, HY: hypothalamus, AQ: cerebral aqueduct, MB: midbrain, CB: cerebellum, 4 V: fourth ventricle, MY: medulla oblongata. **B** Scanning images show ACE2 (green) distribution in the hemisphere sections. **C**–**F** ACE2 (red) ubiquitously distributes in the cerebral microvessels labeled by endothelial cell marker CD31 (green) and pericyte marker PDGFRβ (green), respectively. ACE2 overlapped with pericyte marker PDGFRβ (**F**) but not endothelial cell marker CD31 (**D**). **C** and **E** showed the low magnitude images. **D** and **F** showed the enlarged images of the indicated regions in **C** and **E**, respectively. **G** ACE2 (red) does not distribute in large blood vessels marked by CD31 (green) but is detectable in the meninges wrapping HY. DAPI (blue) was used for nuclear staining. *: indicates large blood vessels. #: indicates meninges. Arrowhead: indicates microvessel. Image magnitude **B**: 10X; **C**, **E** and **G**: 20X; **D** and **F**: 100X

We prepared mouse brain coronal sections representing the main brain regions (Fig. 1A). As shown in Fig. 1B, ACE2 broadly distributed in the brain. To systemically elucidate the expression of ACE2, we grouped the brain regions into four classes: microvascular, parenchymal, ventricular and olfactory. Firstly. we observed that most of the ACE2-positive signals shown in Fig. 1B came from the cerebral microvascular units that ubiquitously distributed throughout the brain. ACE2 strongly expressed in cerebral microvessels marked by the endothelial cell marker CD31 and pericyte marker PDGFRβ (Fig. 1C and E). Cerebral microvessels are comprised by a complete endothelial layer closely paralleled by pericytes to form the vascular components of the BBB. ACE2-positive signals overlapped with the pericyte marker PDGFRβ but not endothelial cells marker CD31, suggesting ACE2 expressed in pericytes but not endothelial cells (Fig. 1D and F). Those data were consistent with previous reports that pericytes instead of endothelial cells expressed ACE2 [50, 51]. On the contrary, there was no detectable ACE2 signal in the large brain blood vessels (Fig. 1G). In addition, the meninges wrapping the hypothalamus (HY) showed strong expression of ACE2 (Fig. 1G).

### Expression of ACE2 in brain parenchymal astrocytes

**Fig. 2:**
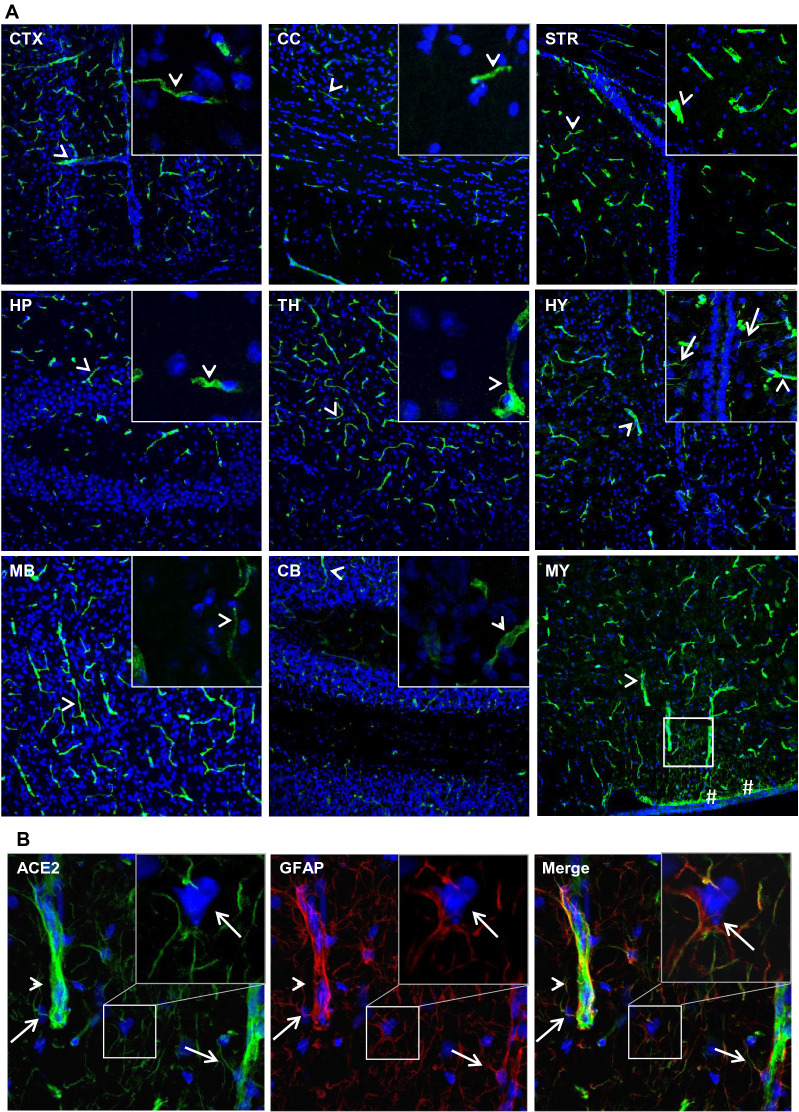

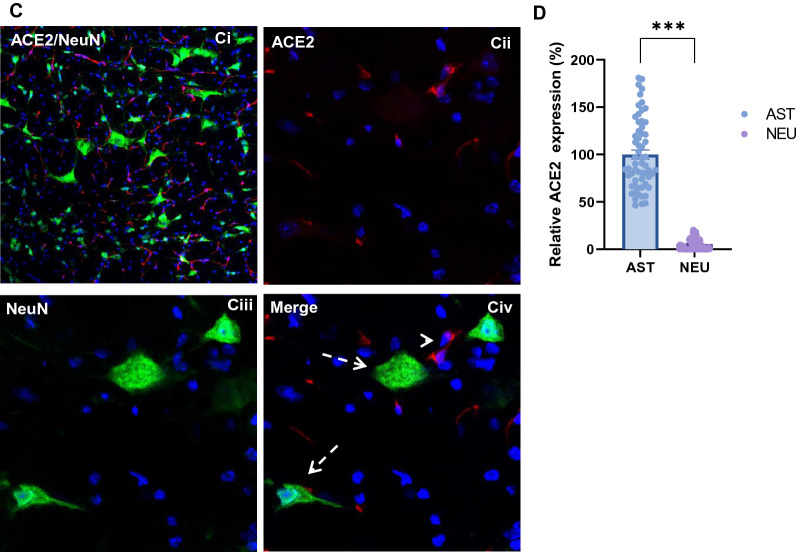
Differential distribution of ACE2 in brain parenchyma. **A** Uneven distribution of ACE2 (green) in indicated brain regions. Each insert shows a comparable high magnitude image of the corresponding region. **B** Enlarged images of the indicated region in the MY panel in A with co-staining of ACE2 (green) and astrocyte marker GFAP (red). **C** Co-staining of ACE2 (red) and neuron marker NeuN (green) in the MY. Cii-Civ show the high magnitude image of the comparable regions of Ci. **D** Quantitation of the relative expression of ACE2 in astrocytes and neurons. Values are presented as mean ± SEM. n = 60. ***: p < 0.001. DAPI (blue) was used for nuclear staining. Arrowhead: indicates microvessel. Arrow: indicates astrocyte. Dash arrow: indicates neuron. #indicates meninges wrapping MY. Image magnitude A and Ci: 20X, B and Cii-iv: 100X

Next, we examined the distribution of ACE2 in different brain parenchymal regions. As shown in Fig. 2A, ACE2 unevenly distributed in the brain parenchyma. High expression of ACE2 was detected in the medulla oblongata (MY) and HY, but low in the cerebral cortex (CTX), corpus callosum (CC), striatum (STR), hippocampus (HP), thalamus (TH), midbrain (MB) and cerebellum (CB), indicating MY and HY were more susceptible to SARS-CoV-2 infection compared to others. Co-staining with cell type-specific markers demonstrated ACE2 primarily overlapped with astrocyte marker GFAP but not the neuronal marker NeuN (Fig. 2B–C). The expression level of ACE2 in astrocytes was dramatically higher than neurons (Fig. 2D), indicating ACE2 predominately expressed in astrocytes instead of neurons in the CNS, which was consistent with previous studies that show astrocytes but not neurons are the main vulnerable sites for SARS-CoV-2 infection [36, 52]. It is worthy to note, some astrocytes directly contacting with the cerebral microvessels expressed ACE2 (Fig. 2B). Besides, the meninges wrapping the MY also showed strong expression of ACE2 (Fig. 2A.MY panel).

### Expression of ACE2 around cerebral ventricles

**Fig. 3:**
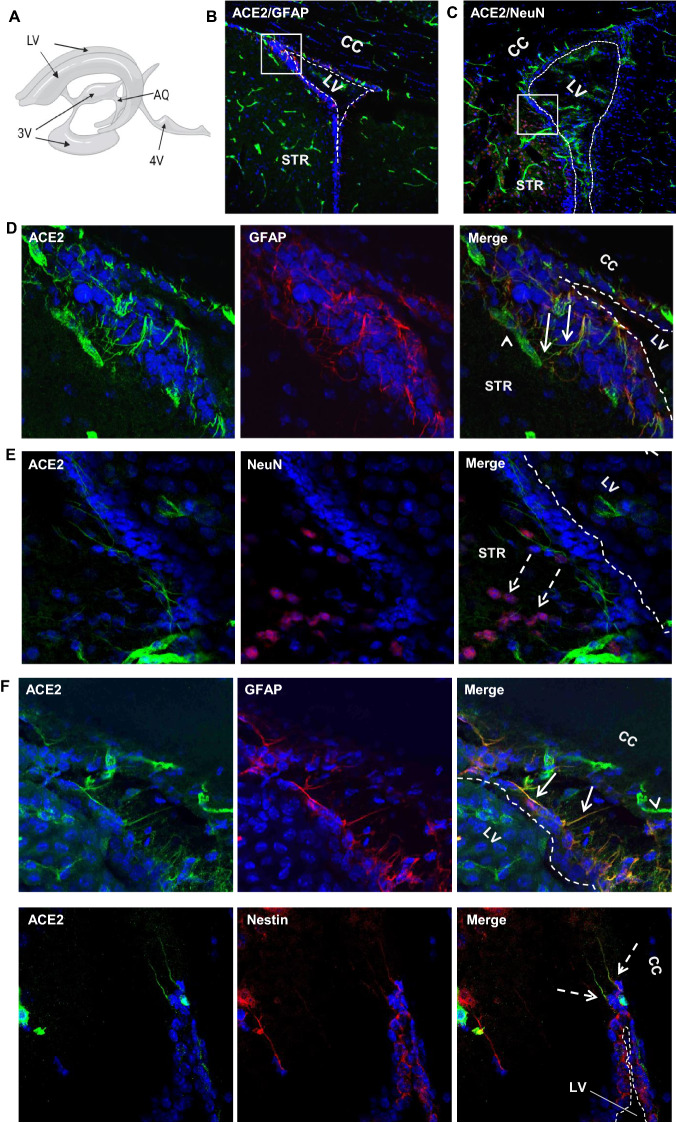

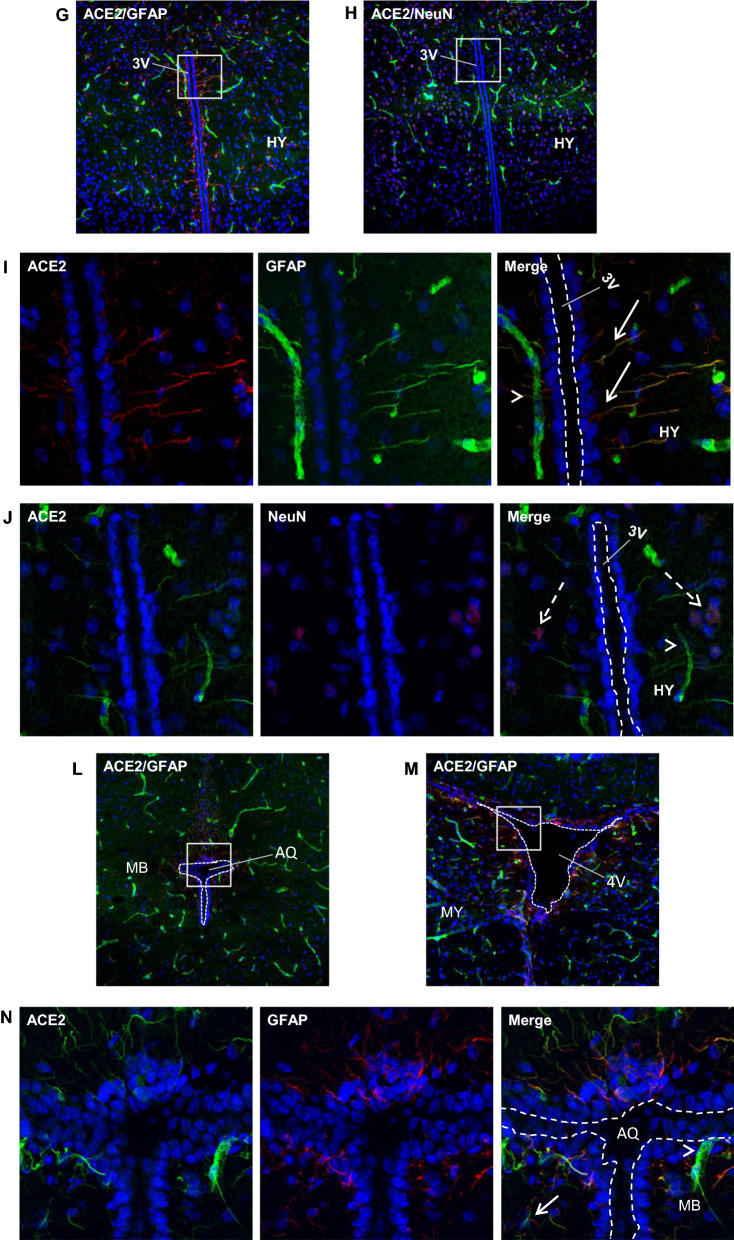

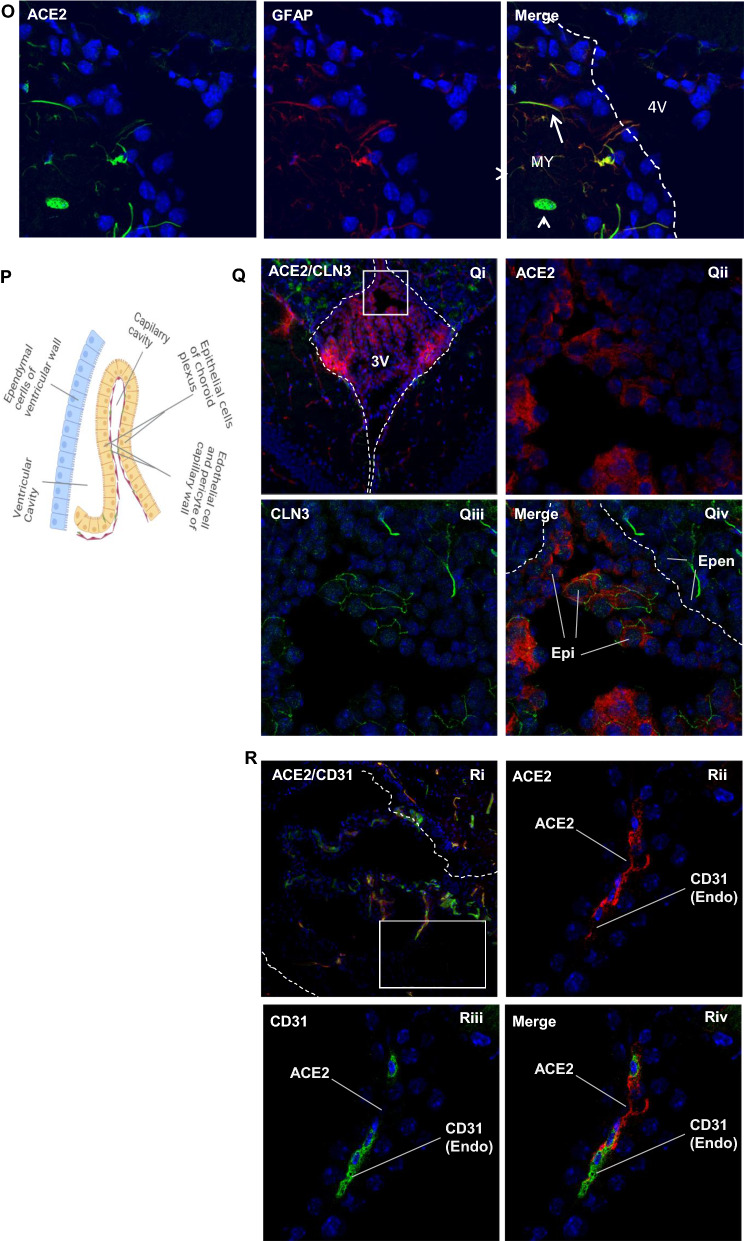
Expression of ACE2 around cerebral ventricles. **A**: Schematic elucidation of the cerebral ventricular system. **B**–**E**: Co-staining of ACE2 (green) with astrocyte marker GFAP (red) or neuron marker NeuN (red) in the subventricular zone (SVZ) of the LV, respectively. **B**–**C** show the low magnitude images. D–E show the enlarged images of the indicated regions in B and C, respectively. Arrow: indicates astrocyte, Dash arrow: indicates neuron. Arrowhead: indicates microvessel. **F**: ACE2 (green) expresses in the GFAP-positive (red) and Nestin-positive (red) radial glial cells in the ventricular zone (VZ) of the LV. Arrow: indicates GFAP-positive radial glial cells. Dash arrow: indicates Nestin-positive radial glial cells. Arrowhead: indicates microvessels. **G–J**: Co-staining of ACE2 (green) with GFAP (red) or NeuN (red) in the 3 V, respectively. G-H show the low magnitude images. I-J show the enlarged images of the indicated regions in G and H, respectively. Arrow: indicates tanycyte, Dash arrow: indicates neuron. Arrowhead: indicates microvessel. **L–O**: Co-staining of ACE2 (green) and GFAP (red) in the AQ and 4 V, respectively. **L**–**M** show the low magnitude images. N–O show the enlarged images of the indicated regions in L and M, respectively. Arrow: indicates astrocyte, Arrowhead: indicates microvessel. **P–R**: Co-staining of ACE2 (red) with ChP epithelial cell marker claudin-3 (CLN3, green) and ChP capillary endothelial cell marker CD31 (green), respectively. P: Schematic elucidation of the structure of ChP in a cerebral ventricle. Q: ACE2 expresses in the ChP epithelial cells in the 3 V. Qii-iv: the enlarged images of the indicated region in Qi. R: ACE2 expresses in the ChP capillaries. Rii-iv: the enlarged images of the indicated region in Ri. DAPI (blue) was used for nuclear staining. ChP: choroid plexus, Epen: ependymal cell of the ventricular wall, Epi: epithelial cell in the ChP, Endo: endothelial cell in the ChP capillaries. The dash lines in all images outline the border of ventricular cavity. Image magnitude B–C, G–H, L–M, Qi and Ri: 20X, D–F, I–J, N–O, Qii-iv and Rii-iv: 100X

We observed ACE2-positive signals around the cerebral ventricular system (Fig. 3A) comprised by the lateral ventricle (LV) (Fig. 3B–C), third ventricle (3 V) (Fig. 3G–H), cerebral aqueduct (AQ) (Fig. 3L) and fourth ventricle (4 V) (Fig. 3M). The data showed ACE2-positive signals primarily overlapped with astrocyte marker GFAP (Fig. 3D and N, O) but not neuron marker NeuN (Fig. 3E and J), suggesting astrocytes but not neurons in those regions were the main cells that expressed ACE2. The ventricle zones (VZ) and subventricular zones (SVZ) of the LV are crucial regions accounting for neurogenesis in the CNS. The GFAP-positive radial glial cells residing in those regions have been known as progenitor cells for generating new neural cells [53–55]. Our data for the first time revealed that the GFAP-positive radial glial cells in the mouse brain expressed ACE2 (Fig. 3F). Co-staining of ACE2 with Nestin, a neural progenitor cell marker, further confirmed that radial glial cells expressed ACE2 (Fig. 3F). Our data indicates those neural progenitor cells could be vulnerable to SARS-CoV-2 infection. In addition, ACE2 was detected in the tanycytes (Fig. 3G and I), which was consistent with a previous study [56]. Tanycytes are a group of GFAP-positive specialized ependymoglial cells lining the 3 V walls with extending processes into the hypothalamus parenchyma [56]. Tanycytes are important for sensing and responding to the fluctuations in whole-body energy status to maintain metabolism homeostasis [57]. Furthermore, we examined ACE2 expression in the choroid plexus (ChP) located in the cerebra ventricular cavities. ChP is formed by projected capillaries lined by fenestrated epithelial layer surrounded by a layer of specialized epithelial cells, and functions as the BCSFB to protect the CNS from unwanted plasma proteins (Fig. 3P). Our data showed ACE2 distribution in the ChP (Fig. 3Q–R). ChP epithelial cells, indicated by the ChP epithelial cell marker claudin-3, showed ACE2 expression (Fig. 3Q). It was worthy to note, ependymal cells lining the ventricular walls did not express ACE2 (Fig. 3Q). Since there are few pericytes in the ChP capillaries, we could not detect ACE2 expression in ChP pericytes. Similar to BBB endothelial cells, ChP capillary endothelial cells, indicated by endothelial cell marker CD31, did not to show detectable ACE2 (Fig. 3R). Therefore, we believe the ACE2 signal around the ChP capillaries could be due to stromal distribution of ACE2 (Fig. 3R). Stromal ACE2 might be secreted by the ChP epithelial cells or be partially from blood flow. The higher permeability of fenestrated capillaries and ACE2-positive epithelial cells make it possible for plasma SAR-CoV-2 and/or the shed S protein from SARS-CoV-2 to enter the CSF. Our data were consistent with previous studies that suggested SARS-CoV-2 might attack the ChP and impair the BCSFB [58, 59].

### Expression of ACE2 along the retrograde olfactory route

**Fig. 4:**
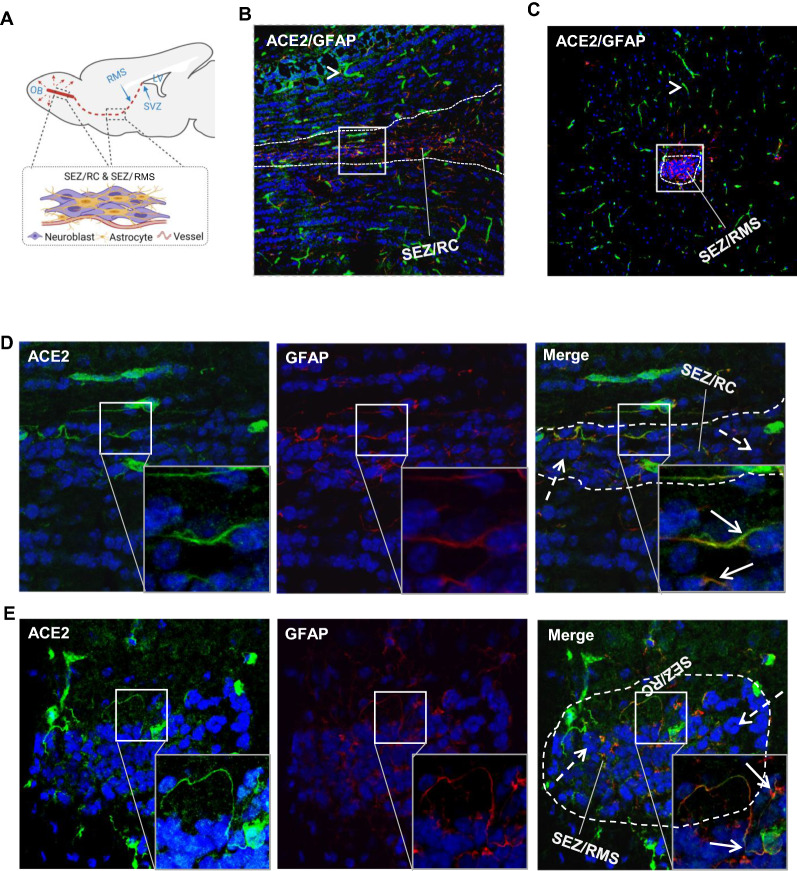
Distribution of ACE2 along the olfactory retrograde route. **A** Schematic elucidation of a gross view of mouse olfactory retrograde route comprised by OB surface, SEZ/RC, SEZ in the rostral migratory stream (SEZ/RMS) and SVZ of the LV (top panel) and a microscopic view of SEZ/RC and SEZ/RMS (bottom panel). **B–E** Co-staining of ACE2 (green) with astrocyte marker GFAP (red) in SEZ/RC and SEZ/RMS. **B**-**C** show the low magnitude images. **D**–**E** show the enlarged images of the indicated regions in **B** and **C**, respectively. DAPI (blue) was used for nuclear staining. Arrow: indicates astrocyte. Dash arrow: indicates neuroblast. Arrowhead: indicates microvessel. The dash lines in all images outline the border of SEZ/RC and SEZ/RMS, respectively. Image magnitude **B**-**C**: 20X, **D**-**E**: 100X

The retrograde olfactory route, by which the neuroblasts migrate from the SVZ of the LV, via SEZ in the rostral migratory stream (SEZ/RMS) and SEZ in the rhinocele (SEZ/RC), to the OB surface (Fig. 4A), has been proposed as a potential route for SARS-CoV-2 entry into the brain. Previous studies have shown the expression of ACE2 in the olfactory epithelial cells [21, 60], implying a possible SARS-CoV-2 entry into the CNS via this route [61]. Whereas it remains unclear if the viruses can migrate along the SEZ/RMS and SEZ/ RC sites. Our data showed ACE2 expression in the astrocytes located in the SEZ/RC (Fig. 4B and D) and SEZ/RMS (Fig. 4C and E), providing further evidence to support the retrograde olfactory route as a possible route for SARS-CoV-2 entry. Similar as the mature neurons, ACE2 was rarely detectable in the neuroblasts (Fig. 4D–E).

### Expression of ACE2 in primary cultured brain cells

**Fig. 5:**
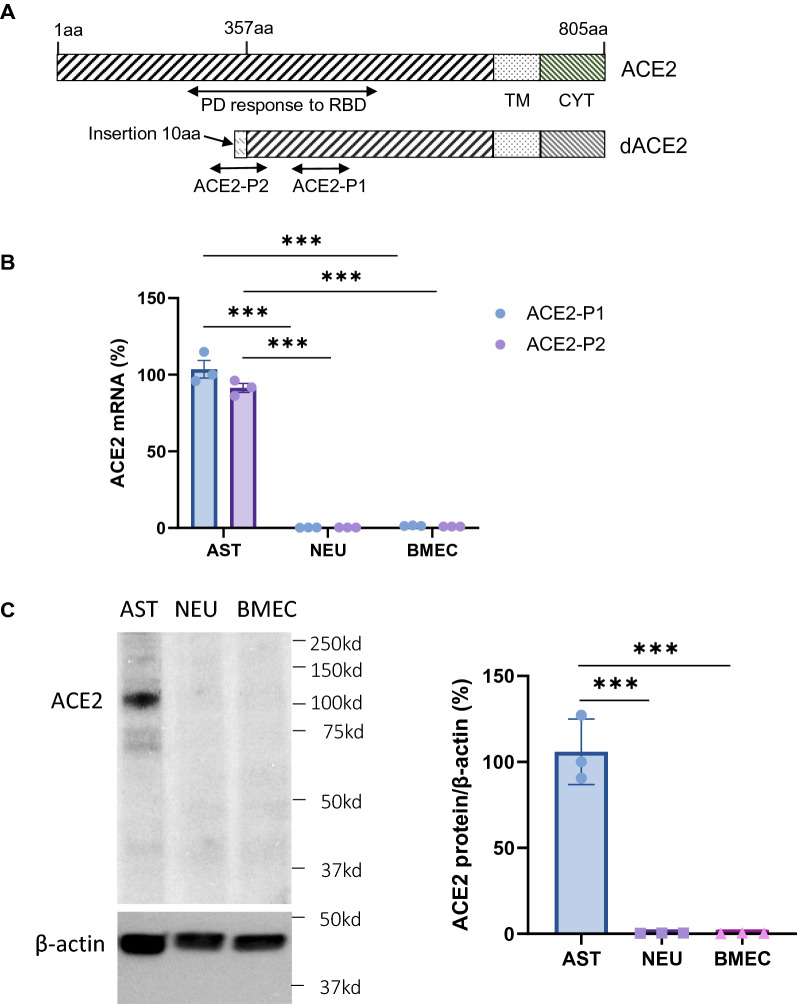
Expression of ACE2 in primary cultured brain cells. **A** Schematic elucidation of the structure of ACE2 protein. PD: peptidase domain, RBD: receptor-binding domain, TM: transmembrane domain, CYT: cytosolic domain, dACE2: truncated ACE2. ACE2-P1 or ACE2-P2: isoform-specific primers designed for detecting both full-length and dACE2 or the full-length ACE2 only, respectively. aa: amino acid. **B**–**C** Real-time PCR detection of ACE2 mRNA expression (**B**) and western blot detection of ACE2 protein expression (**C**) in astrocytes (AST), neurons (NEU) and brain microvascular endothelial cells (BMEC). Real-time PCR and western blot assays were done in triplicate. Values are presented as mean ± SEM. n = 3. ***: p < 0.001

To further validate the cell-type specific expression of ACE2, we isolated primary astrocytes, neurons and BMECs to examine the ACE2 expression in those cells. To maximally maintain the natural property of the primary cells, all primary cells used this study were passaged no more than one time. Two recent studies reveal a new truncated isoform of ACE2 (dACE2) in the respiratory epithelia [62, 63]. The dACE2 has a short N-terminal domain lacking the PD responsible for interacting with SARS-CoV-2’s RBD (Fig. 5A), thus cannot facilitate SAR-CoV-2 to enter into the host cells [62, 63]. It raises a question which isoform(s) of ACE2 express in astrocytes? To answer that, we designed two pairs of primers targeting the full-length ACE2 only or both full-length ACE2 and dACE2, respectively. Our data showed that more than 90% of ACE2 in astrocytes was the full-length ACE2 (Fig. 5B), indicating astrocytes are susceptible to SARS-Cov-2 infection. Similar to the results in brain sections, ACE2 expression in primary astrocytes was dramatically higher than neuron and BMECs at both mRNA and protein levels (Fig. 5B, C).

### A pre-conditioned neuroinflammatory state induced by smoking

**Fig. 6:**
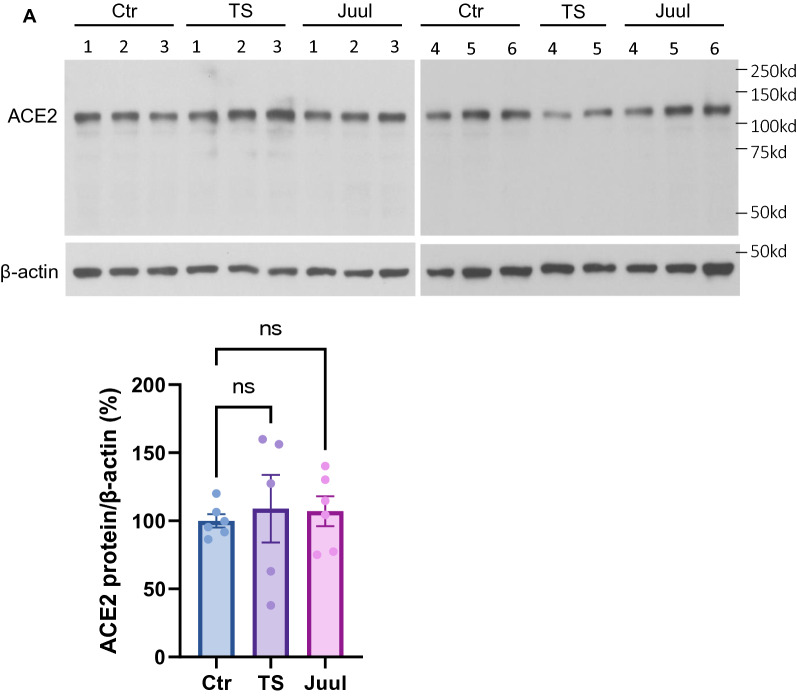

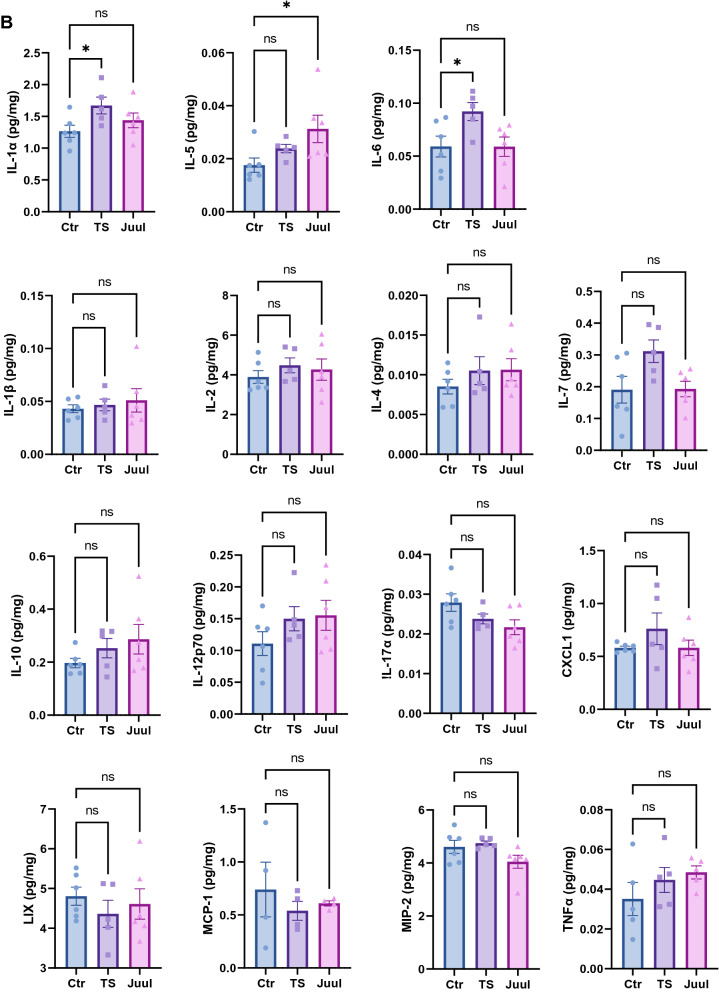
Smoking altered neuroinflammatory and immunomodulatory cytokines in brain without significantly affecting ACE2 expression. **A**: Western blot detection of ACE2 expression in mouse brains exposed to experimental smoking. Top panel shows the western blot images. Bottom panel shows the quantitation of band intensity of the top panel. Ctr, TS and Juul: control, tobacco smoking and Juul electronic nicotine vaping group, respectively. **B**: Altered cytokines in the brains induced by experimental smoking exposure. Values are presented as mean ± SEM. n = 5–6. *: p < 0.05. ns: not statistically significant

A larger number of studies have linked the risk factor of smoking to COVID-19 severity in patients. Previous studies demonstrate smoking upregulates pulmonary ACE2 expression that has been considered to attribute to worsened outcomes in smokers [40–42]. Whereas it is not clear if smoking exacerbates neural pathogenesis in a similar or alternative manner. It is also not clear if electronic nicotine vaping would harm the lungs or CNS in a similar manner to tobacco smoking. In this study, we exposed mice with experimental smoking including both tobacco smoking and electronic nicotine vaping to evaluate brain ACE2 expression. A sub-chronic (14 days) exposure was used to mimic the smoking behavior of a current smoker [64, 65]. Our data revealed that experimental smoking did not significantly alter ACE2 expression in the smoking-exposed brains compared to the control (Fig. 6A). Then, we performed a cytokine screening of the smoking-exposed brain tissues. The data revealed that proinflammatory cytokines interleukin (IL)-1α, IL-6 and immunomodulator IL-5 were significantly upregulated in the tobacco smoke- and/or electronic nicotine vapor-exposed brains compared to the control (Fig. 6B). The cytokines IL-7, IL-10, IL-12p70 and TNFα also showed an increased trend and IL-17α and LIX showed a deceased trend, although they were not statistically significant (Fig. 6B). Taken together, these data indicate that smoking may interplay with SARS-CoV-2 infection to exacerbate COVID-19 brain symptoms by pre-conditioning the brain into a neuroinflammatory state.

## Discussion

Accumulating evidence shows that the CNS could serve as a vulnerable site for neuroinflammation and infection in COVID-19 patients. Postmortem examinations demonstrate a neuroinflammatory signature in infected brain tissues [9, 66]. The neuroinflammation could be a consequence of direct intracranial viral infection or secondary impacts from extracranially viral infection leading to systematic hypoxia and blood–brain barrier breakdown or excessive inflammatory factors that penetrate into the CNS [22]. In patients with severe cardio-pulmonary disorders and/or respiratory failure, neurological manifestations could be largely due to inadequate supply of oxygenated blood to the brain, particularly in patients without positive SARS-CoV-2 in CNS. However, the detection of positive SARS-CoV-2 in CSF of certain patients with mild symptoms and lacking significant cardio-pulmonary manifestations implies a possible neural invasion in these patients [13–19]. The observation of positive SARS-CoV-2 signal not coinciding with immune cell infiltration in postmortem brain tissues also suggests that SARS-CoV-2-related neurological complications could be the direct consequence of the neurovirulent properties of SARS-CoV-2 [22, 67]. The current study shows a spatial- and cell type-specific distribution of ACE2 in the brain, indicating certain brain sites and cell types are more susceptible to SARS-CoV-2 infection than the others. Deregulation of ACE2 signaling and ACE2-expressing cells in specific brain regions might impair various brain functions and therefore be associated with specific symptoms in COVID-19 patients. For example, impairment of ACE2-expressing astrocytes in the olfactory regions might be associated with the loss of sense of smell and taste (Fig. 4), while impairment in the medulla oblongata might be related to pulmonary and cardiac disorders since this region contains vital respiratory and cardiac regulatory centers (Fig. 2A.MY panel and 2B). Impairment in the hypothalamus, including tanycytes of the 3 V, might be associated with systemic disorders such as thrombosis, immune-inflammation and cardiorespiratory aberrancy since hypothalamus is responsible for sensing and regulating the whole-body fluctuation in fluid, electrolyte, metabolism and immunomodulation in addition to being connected to the olfactory/gustative and brainstem cardiorespiratory centers (Fig. 2A.HY panel, 3G and 3I) [56, 68–70]. Lastly, modified ACE2 signaling in pericytes and astrocytes around microvessels might be associated with cerebral microvascular disorders in COVID19 patients, such as possible BBB damage and stroke, since these cells play essential roles in regulating microvascular integrity and function as well as maintaining CNS homeostasis (Fig. 1F and 2B). Furthermore, our study, for the first time, reveals that ACE2 might express in the radial glial cells (Fig. 3F) in the VZ and SVZ of the LV that have been known as progenitor cells in the CNS [53–55]. Impairment of those progenitor cells, as well as astrocytes residing in the neurogenesis zones, might contribute to the lasting post-infection neuropsychological disorders in recovering patients. Consistent with our data, a recent study with human brain organoids reports that neural progenitor cells are vulnerable to SARS-CoV-2 infection [71]. Furthermore, Klempin et al. report ACE2 plays an essential role in neurogenesis [72]. Their study shows ACE2-deficent mice demonstrate impaired exercise-stimulating neurogenesis capacity through a mechanism independent of the RAS and serotonin pathways. Our finding of the intrinsic expression of ACE2 in the radial glial cells might provide a plausible explanation to their study. The ACE2 expression in the brain endothelial cells remains controversial. Although early studies show ACE2 expression in vascular endothelial cells in the human brain, more recent studies demonstrated ACE2 is not expressed in the vascular endothelial cells but instead in pericytes [11, 35]. These inconsistencies could be due the differences in the detection methods, sample preparation, technique limitation in different labs, and possibly altered ACE2 expression by some pre-existed health issues. Our study shows that ACE2 expression is in brain microvascular pericytes instead of endothelial cells (Fig. 1D, F-G). Consistently, our data also show ACE2 is most likely distributed in the ChP stroma tissues instead of ChP capillary endothelial cells (Fig. 3R). The ChP stroma ACE2 could be secreted by local ChP epithelial cell or be filtrated from the blood flow because of the high permeability of ChP capillaries. The ChP stromal ACE2 raises a potential role of extracellular ACE2 in COVID-19, which needs further investigations.

Our studies show that astrocytes are the predominant cells that express the full-length ACE2 (Fig. 5), suggesting those cells are indeed susceptible to possible SARS-CoV-2 infection. Recent studies with postmortem brain tissues demonstrate astrocytes are the major SARS-CoV-2 infected cells, but neurons are rarely infected [36, 52]. It has also been reported that CNS injuries are accompanied by an elevated plasma level of GFAP in moderate and severe COVID-19 patients [73]. Our data, together with previous studies, highlight a potential role of astrocyte injury in the CNS transmission and pathogenesis of COVID-19. As one of the most abundant cell types in the CNS, astrocytes perform a large number of functions, such as maintaining CNS homeostasis via secreting neurotrophic factors and regulating intracranial fluid, ionic and metabolic balance, supporting and modulating neurons, regulating BBB integrity and function, and modulating CNS inflammatory and immune responses to physical, pathological and infectious insults [22–24]. It is worthy to note, as an essential counter actor in Ang II signaling, ACE2 protects multiple tissues and organs from inflammatory injuries [29]. SARS-CoV-2 infection of astrocytes could simultaneously impair the protective functions of ACE2 signaling and astrocytes in the CNS. The interplay of ACE2 signaling and astrocytes might play a key role in any neurovascular impairment due to COVID-19, since these cells are in close proximity to brain pericyte and endothelial cells as well as play an indispensable role in the regulation of the neurovascular unit (Fig. 2B).

Several potential brain entry routes for SARS-CoV-2 have been proposed. Crossing BBB is one of the potential routes. Our data show ACE2 expression in cerebral endothelial cells is extreme low, even lower than neurons (Fig. 5B–C). Given that neurons were rarely infected by SARS-CoV-2 [36, 52], endothelial cells might be unsusceptible to SARS-CoV-2 infection, too. Thus, it is less likely for SARS-CoV-2 in the blood to enter the CNS in patients through an intact BBB. Crossing BCSFB located in the cerebral ventricular ChP is another potential route. ChP is formed by projected fenestrated capillaries surrounded by a layer of epithelial cells in the cerebral ventricular cavities. Fenestrated capillaries in this region lead to a higher permeability observed in the BCSFB compared to BBB [74]. Our data suggest ACE2 expression in ChP epithelial cells and stromal tissues between the ChP epithelial cells and the ChP capillaries (Fig. 3Q–R), suggesting possible SARS-CoV-2 filtrated from the fenestrated capillaries may infect ChP epithelial cells and impact BCSFB integrity followed by movement into the CSF. Yet, ACE2 is undetectable in the ependymal cells lining the ventricular walls (Fig. 3Q), indicating it might be less likely for SARS-CoV-2 in the CSF to enter the brain parenchyma directly through the ventricular walls where there is a complete ependymal lining. However, it is possible that, in some regions of the ventricular walls, ACE2-positive cells in the SVZ regions could penetrate through the ependymal layer and contact the CSF. Also, tanycytes located at the 3 V walls in the hypothalamus (Fig. 3G and I) directly contact CSF. These cells that directly contact both the CSF and brain parenchyma might be susceptible sites for SARS-CoV-2 entry. Furthermore, the viruses may spread in the CSF and infect certain regions of meninges such as the ACE2-expressing meninges wrapping the hypothalamus and medulla oblongata to enter the brain parenchyma (Fig. 1G and 2A.MY panel). In line with this hypothesis, medulla oblongata and hypothalamus have been reported as the venerable sites for SARS-CoV-2 infection [9, 56, 67, 75]. In addition, impaired ChP could induce BCSFB damage leading to the leakage of unwanted plasma proteins into the CSF, which has been observed in more than 40% of patients [58, 76]. Our data together with other studies support crossing BCSFB might be a likely route for SARS-CoV-2 entry. Retrograde olfactory migration is another possible route. Previous studies have demonstrated the presentence of ACE2 and SARS-CoV-2 in the OB epithelial cells [21, 60, 61], but it remains unclear if SARS-CoV-2 can migrate into the brain along this route. Our data show ACE2 expresses in astrocytes residing in the subependymal zone (SEZ)/RC and SEZ/RMS sites, suggesting SARS-CoV-2 could be transported into the CNS by those astrocytes along this retrograde olfactory route (Fig. 4).

Several risk factors, including smoking, have been reported to be associated with disease severity in COVID-19 patients [38, 39]. Smoking, including tobacco smoking and electronic nicotine vaping, upregulates pulmonary ACE2 expression which could attribute to worsened outcomes observed in smokers [40–42]. Smoking might worsen COVID-19 by pre-positioning an abnormal neuroinflammatory/immune state, attenuating the inflammatory regulatory capacity, reducing the pathogen clearance capacity, facilitating virus entry by impairing BBB, and/or might facilitate SARS-CoV-2 replication as it facilitates human immunodeficiency virus (HIV) replication in the host cells [77]. However, it is not clear if smoking exacerbates neuropathogenesis in a similar or alternative manner. It is also not clear if electronic nicotine vaping would harm the lungs or CNS in a similar manner to tobacco smoking. Our data show experimental exposure to tobacco smoke or electronic nicotine vapor for 14 days does not significantly alter ACE2 expression in the mouse brain tissue (mRNA or protein) (Fig. 6A). These data suggest that unlike the pulmonary system, where smoking upregulates ACE2 levels, smoking might not directly contribute to the SARS-CoV-2/ACE2 interaction-mediated viral entry and/or pathological effects in the brain [40–42]. Instead, it upregulates several cytokines such as IL-1α, IL-6 for tobacco smoking and IL-5 for electronic nicotine vaping (Fig. 6B). These cytokines have been well-documented to be closely related to COVID-19 severity [78–83]. Many studies have pointed to IL-6 as a crucial signature of the cytokine storm in severe COVID-19 patients [79–84]. Members of IL-1 family are cardinal mediators of inflammation [85], among which IL-1α is one of the archetypical proinflammatory cytokines. IL-5 is a type 2 cytokine functioning as an immunomodulator, upregulation of which might compromise the early immune defense against infection [86]. Consistent with our data, previous studies have shown smoking increases IL-1α and IL-6 in the plasma and brain tissues [87–90]. Our laboratory has also shown smoking exposure (both tobacco smoke and electronic nicotine vaping) is associated with aggravated brain edema as well as reduced antioxidative molecule NRF2 and increased proinflammatory cytokine TNFα in mouse brain under normoxic and/or ischemic stroke conditions [49, 91]. It is worthy to note, activated astrocytes are the predominant source of IL-6 and IL-1α, the key contributors to neuroinflammation in the CNS [22, 85, 92]. Taken together, our ACE2 brain distribution data, alongside studies suggesting smoking may pre-condition a proinflammatory scenario in the brain, suggest that the CNS could be more susceptible to infectious insults with smoking, including SARS-CoV-2 infection, which could be partly due to astrocytes.

There are a few potential weaknesses in the current study design. A sub-chronic (14 days) smoking exposure was used to mimic the effect of tobacco and nicotine exposure on the brain, and the data shows this does not significantly alter brain ACE2 levels. However, the effect of long-term smoking on brain ACE2 levels remains unclear. A chronic exposure (> 1 month or longer) should be completed to address this question in the future. In addition, young and healthy mice were investigated in the current study. Since COVID-19 severity has been well-documented to be correlated with several pre-existed health issues such as diabetes, obesity and hypertension [93], future studies are warranted to investigates the potential role of ACE2 signaling in the neural transmission and pathogenesis of COVID-19 under these pre-existing disease states. In the future, it would also be important to test ACE2 signaling in aged mice.

## Conclusions

The present study demonstrates a spatial- and cell type-specific expression of ACE2 in the brain. Results indicate certain sites (medulla oblongata, hypothalamus, choroid plexus, and retrograde olfactory route) and cell types (astrocyte, pericyte, radial glial cell, tanycyte, and choroid plexus epithelial cell) are more vulnerable to SARS-CoV-2 infection, highlighting a potential role of astrocyte ACE2 in the neural transmission and pathogenesis of COVID-19. These data should help to explain the acute and lasting post-infection neuropsychological manifestations in COVID-19 patients. Our study also suggests a pre-conditioned neuroinflammatory and immunocompromised scenario might attribute to the exacerbated COVID-19 severity in the smokers.

## Data Availability

All data generated or analyzed during this study are included in the published article.

## Abbreviations

ACE2: Angiotensin converting enzyme 2
RAS: Renin-angiotensin system
CNS: Central neural system
BBB: Blood brain barrier
CSF: Cerebrospinal fluid
BCSFB: Blood-CSF barrier
PASC: Post-acute sequelae of SARS-CoV-2 infection
OB: olfactory bulb
CTX: Cerebral cortex
CC: Corpus callosum
STR: striatum
LV: Lateral ventricle
HP: Hippocampus
TH: Thalamus
3 V: Third ventricle
HY: Hypothalamus
AQ: Cerebral aqueduct
MB: Midbrain
CB: Cerebellum
4 V: Fourth ventricle
MY: Medulla oblongata
SVZ: Subventricular zone
SEZ: Subependymal zone
SEZ/RC: SEZ in the rhinocele
SEZ/RMS: SEZ in the rostral migratory stream

## References

[1] Iadecola C, Anrather J, Kamel H (2020). Effects of COVID-19 on the nervous system. Cell.

[2] Kumar D, Jahan S, Khan A, Siddiqui AJ, Redhu NS, Wahajuddin, Khan J, Banwas S, Alshehri B, Alaidarous M (2021). Neurological manifestation of SARS-CoV-2 induced inflammation and possible therapeutic strategies against COVID-19. Mol Neurobiol.

[3] Mao L, Jin H, Wang M, Hu Y, Chen S, He Q, Chang J, Hong C, Zhou Y, Wang D (2020). Neurologic manifestations of hospitalized patients with coronavirus disease 2019 in Wuhan China. JAMA Neurol.

[4] Helms J, Kremer S, Merdji H, Clere-Jehl R, Schenck M, Kummerlen C, Collange O, Boulay C, Fafi-Kremer S, Ohana M (2020). Neurologic features in severe SARS-CoV-2 Infection. N Engl J Med.

[5] Nagu P, Parashar A, Behl T, Mehta V (2021). CNS implications of COVID-19: a comprehensive review. Rev Neurosci.

[6] Vaira LA, Calvo-Henriquez C, Mayo-Yanes M, Hoch CC, Lechien JR (2021). Olfactory and gustatory dysfunctions are difficult to evaluate in hospitalized COVID-19 patients. Am J Otolaryngol.

[7] Nuzzo D, Vasto S, Scalisi L, Cottone S, Cambula G, Rizzo M, Giacomazza D, Picone P (2021). Post-acute COVID-19 neurological syndrome: a new medical challenge. J Clin Med.

[8] Moghimi N, Di Napoli M, Biller J, Siegler JE, Shekhar R, McCullough LD, Harkins MS, Hong E, Alaouieh DA, Mansueto G (2021). The Neurological manifestations of post-acute sequelae of SARS-CoV-2 infection. Curr Neurol Neurosci Rep.

[9] Matschke J, Lutgehetmann M, Hagel C, Sperhake JP, Schroder AS, Edler C, Mushumba H, Fitzek A, Allweiss L, Dandri M (2020). Neuropathology of patients with COVID-19 in Germany: a post-mortem case series. Lancet Neurol.

[10] Remmelink M, De Mendonca R, D'Haene N, De Clercq S, Verocq C, Lebrun L, Lavis P, Racu ML, Trepant AL, Maris C (2020). Unspecific post-mortem findings despite multiorgan viral spread in COVID-19 patients. Crit Care.

[11] Bocci M, Oudenaarden C, Saenz-Sarda X, Simren J, Eden A, Sjolund J, Moller C, Gisslen M, Zetterberg H, Englund E (2021). Infection of brain pericytes underlying neuropathology of COVID-19 patients. Int J Mol Sci.

[12] Huang YH, Jiang D, Huang JT (2020). SARS-CoV-2 detected in cerebrospinal fluid by PCR in a case of COVID-19 encephalitis. Brain Behav Immun.

[13] de Freitas GR, Figueiredo MR, Vianna A, Brandao CO, Torres-Filho HM, Martins AFA, Tovar-Moll F, Barroso PF (2021). Clinical and radiological features of severe acute respiratory syndrome coronavirus 2 meningo-encephalitis. Eur J Neurol.

[14] Allahyari F, Hosseinzadeh R, Nejad JH, Heiat M, Ranjbar R (2021). A case report of simultaneous autoimmune and COVID-19 encephalitis. J Neurovirol.

[15] Domingues RB, Mendes-Correa MC, de Moura Leite FBV, Sabino EC, Salarini DZ, Claro I, Santos DW, de Jesus JG, Ferreira NE, Romano CM (2020). First case of SARS-COV-2 sequencing in cerebrospinal fluid of a patient with suspected demyelinating disease. J Neurol.

[16] Glavin D, Kelly D, Wood GK, McCausland BM, Ellul MA, Varatharaj A, Galea I, Thomas RH, Michael BD, Gallen B (2021). COVID-19 encephalitis with SARS-CoV-2 detected in cerebrospinal fluid presenting as a stroke mimic. J Stroke Cerebrovasc Dis.

[17] Krueger MB, Montenegro RC, de Araujo Coimbra PP, de Queiroz LL, Fiorenza RM, da Silva Fernandes CJ, Pessoa MSL, Rodrigues CL, da Cruz CG, de Araujo VV (2021). A wide spectrum of neurological manifestations in pediatrics patients with the COVID-19 infection: a case series. J Neurovirol.

[18] Luis MB, Liguori NF, Lopez PA, Alonso R (2021). SARS-CoV-2 RNA detection in cerebrospinal fluid: presentation of two cases and review of literature. Brain Behav Immun Health.

[19] Moriguchi T, Harii N, Goto J, Harada D, Sugawara H, Takamino J, Ueno M, Sakata H, Kondo K, Myose N (2020). A first case of meningitis/encephalitis associated with SARS-Coronavirus-2. Int J Infect Dis.

[20] Lee MH, Perl DP, Nair G, Li W, Maric D, Murray H, Dodd SJ, Koretsky AP, Watts JA, Cheung V (2021). Microvascular injury in the brains of patients with Covid-19. N Engl J Med.

[21] Ye Q, Zhou J, He Q, Li RT, Yang G, Zhang Y, Wu SJ, Chen Q, Shi JH, Zhang RR (2021). SARS-CoV-2 infection in the mouse olfactory system. Cell Discov.

[22] Tavcar P, Potokar M, Kolenc M, Korva M, Avsic-Zupanc T, Zorec R, Jorgacevski J (2021). Neurotropic viruses, astrocytes, and COVID-19. Front Cell Neurosci.

[23] Vargas G, Medeiros Geraldo LH, Gedeao Salomao N, Viana Paes M, ReginaSouzaLima F, CarvalhoAlcantaraGomes F (2020). Severe acute respiratory syndrome coronavirus 2 (SARS-CoV-2) and glial cells: Insights and perspectives. Brain Behav Immun Health.

[24] Tremblay ME, Madore C, Bordeleau M, Tian L, Verkhratsky A (2020). Neuropathobiology of COVID-19: the role for Glia. Front Cell Neurosci.

[25] Hoffmann M, Kleine-Weber H, Schroeder S, Kruger N, Herrler T, Erichsen S, Schiergens TS, Herrler G, Wu NH, Nitsche A (2020). SARS-CoV-2 cell entry depends on ACE2 and TMPRSS2 and is blocked by a clinically proven protease inhibitor. Cell.

[26] Wan Y, Shang J, Graham R, Baric RS, Li F (2020). Receptor recognition by the novel coronavirus from Wuhan: an analysis based on decade-long structural studies of SARS coronavirus. J Virol.

[27] Jackson CB, Farzan M, Chen B, Choe H (2022). Mechanisms of SARS-CoV-2 entry into cells. Nat Rev Mol Cell Biol.

[28] Shang J, Wan Y, Luo C, Ye G, Geng Q, Auerbach A, Li F (2020). Cell entry mechanisms of SARS-CoV-2. Proc Natl Acad Sci USA.

[29] Gheblawi M, Wang K, Viveiros A, Nguyen Q, Zhong JC, Turner AJ, Raizada MK, Grant MB, Oudit GY (2020). Angiotensin-converting enzyme 2: SARS-CoV-2 receptor and regulator of the renin-angiotensin system: celebrating the 20th anniversary of the discovery of ACE2. Circ Res.

[30] Hemnes AR, Rathinasabapathy A, Austin EA, Brittain EL, Carrier EJ, Chen X, Fessel JP, Fike CD, Fong P, Fortune N (2018). A potential therapeutic role for angiotensin-converting enzyme 2 in human pulmonary arterial hypertension. Eur Respir J..

[31] Khan A, Benthin C, Zeno B, Albertson TE, Boyd J, Christie JD, Hall R, Poirier G, Ronco JJ, Tidswell M (2017). A pilot clinical trial of recombinant human angiotensin-converting enzyme 2 in acute respiratory distress syndrome. Crit Care.

[32] Liu Y, Yang Y, Zhang C, Huang F, Wang F, Yuan J, Wang Z, Li J, Li J, Feng C (2020). Clinical and biochemical indexes from 2019-nCoV infected patients linked to viral loads and lung injury. Sci China Life Sci.

[33] Hernandez VS, Zetter MA, Guerra EC, Hernandez-Araiza I, Karuzin N, Hernandez-Perez OR, Eiden LE, Zhang L (2021). ACE2 expression in rat brain: implications for COVID-19 associated neurological manifestations. Exp Neurol.

[34] Torices S, Cabrera R, Stangis M, Naranjo O, Fattakhov N, Teglas T, Adesse D, Toborek M (2021). Expression of SARS-CoV-2-related receptors in cells of the neurovascular unit: implications for HIV-1 infection. J Neuroinflammation.

[35] Bodnar B, Patel K, Ho W, Luo JJ, Hu W (2021). Cellular mechanisms underlying neurological/neuropsychiatric manifestations of COVID-19. J Med Virol.

[36] Andrews MG, Mukhtar T, Eze UC, Simoneau CR, Perez Y, Mostajo-Radji MA, Wang S, Velmeshev D, Salma J, Kumar GR (2021). Tropism of SARS-CoV-2 for developing human cortical astrocytes. bioRxiv.

[37] Zou X, Chen K, Zou J, Han P, Hao J, Han Z (2020). Single-cell RNA-seq data analysis on the receptor ACE2 expression reveals the potential risk of different human organs vulnerable to 2019-nCoV infection. Front Med.

[38] Sifat AE, Nozohouri S, Villalba H, Vaidya B, Abbruscato TJ (2020). The role of smoking and nicotine in the transmission and pathogenesis of COVID-19. J Pharmacol Exp Ther.

[39] Archie SR, Cucullo L (2020). Cerebrovascular and neurological dysfunction under the threat of COVID-19: is there a comorbid role for smoking and vaping?. Int J Mol Sci.

[40] Cai G, Bosse Y, Xiao F, Kheradmand F, Amos CI (2020). Tobacco smoking increases the lung gene expression of ACE2, the receptor of SARS-CoV-2. Am J Respir Crit Care Med.

[41] Lallai V, Manca L, Fowler CD (2021). E-cigarette vape and lung ACE2 expression: implications for coronavirus vulnerability. Environ Toxicol Pharmacol.

[42] Smith JC, Sausville EL, Girish V, Yuan ML, Vasudevan A, John KM, Sheltzer JM (2020). Cigarette smoke exposure and inflammatory signaling increase the expression of the SARS-CoV-2 receptor ACE2 in the respiratory tract. Dev Cell.

[43] Jennifer V, Welser-Alves AB, Milner R (2014). Isolation and culture of primary mouse brain endothelial cells. Cerebral Angiogenesis Methods Protocols Methods Mol Biol.

[44] Sifat AE, Nozohouri S, Villalba H, Al Shoyaib A, Vaidya B, Karamyan VT, Abbruscato T (2020). Prenatal electronic cigarette exposure decreases brain glucose utilization and worsens outcome in offspring hypoxic-ischemic brain injury. J Neurochem.

[45] Nozohouri S, Zhang Y, Albekairi TH, Vaidya B, Abbruscato TJ (2021). Glutamate buffering capacity and blood-brain barrier protection of opioid receptor agonists biphalin and nociceptin. J Pharmacol Exp Ther.

[46] Zhang Y, Dong Y, Melkus MW, Yin S, Tang SN, Jiang P, Pramanik K, Wu W, Kim S, Ye M (2018). Role of P53-senescence induction in suppression of LNCaP prostate cancer growth by cardiotonic compound Bufalin. Mol Cancer Ther.

[47] Zhang Y, Won SH, Jiang C, Lee HJ, Jeong SJ, Lee EO, Zhang J, Ye M, Kim SH, Lu J (2012). Tanshinones from Chinese medicinal herb Danshen (Salvia miltiorrhiza Bunge) suppress prostate cancer growth and androgen receptor signaling. Pharm Res.

[48] Zhang Y, Kim KH, Zhang W, Guo Y, Kim SH, Lu J (2012). Galbanic acid decreases androgen receptor abundance and signaling and induces G1 arrest in prostate cancer cells. Int J Cancer.

[49] Kaisar MA, Villalba H, Prasad S, Liles T, Sifat AE, Sajja RK, Abbruscato TJ, Cucullo L (2017). Offsetting the impact of smoking and e-cigarette vaping on the cerebrovascular system and stroke injury: Is Metformin a viable countermeasure?. Redox Biol.

[50] He LMM, Muhl L, Sun Y, Pietilä R, Nahar K (2020). Pericyte-specific vascular expression of SARS-CoV-2 receptor ACE2—implications for microvascular inflammation and hypercoagulopathy in COVID-19. bioRxiv.

[51] Hirunpattarasilp JGC, Freitas F, Sethi H, Kittler JT, Huo J, RaymondOwens J, Attwell D (2021). SARS-CoV-2 binding to ACE2 triggers pericyte-mediated angiotensin-evoked cerebral capillary constriction. bioRxiv.

[52] Crunfli FCV, Veras FP, Vendramini PH, Valença AGF, Antunes ASLM (2021). SARS-CoV-2 infects brain astrocytes of COVID-19 patients and impairs neuronal viability. medRxiv.

[53] Merkle FT, Tramontin AD, Garcia-Verdugo JM, Alvarez-Buylla A (2004). Radial glia give rise to adult neural stem cells in the subventricular zone. Proc Natl Acad Sci USA.

[54] Noctor SC, Martinez-Cerdeno V, Ivic L, Kriegstein AR (2004). Cortical neurons arise in symmetric and asymmetric division zones and migrate through specific phases. Nat Neurosci.

[55] Johnson K, Barragan J, Bashiruddin S, Smith CJ, Tyrrell C, Parsons MJ, Doris R, Kucenas S, Downes GB, Velez CM (2016). Gfap-positive radial glial cells are an essential progenitor population for later-born neurons and glia in the zebrafish spinal cord. Glia.

[56] Nampoothiri SSF, Ternier G, Fernandois D, Coelho C, Imbernon M, Deligia E, Perbet R, Florent V, Baroncini M (2020). The hypothalamus as a hub for SARS-CoV-2 brain infection and pathogenesis. bioRxiv.

[57] Lhomme T, Clasadonte J, Imbernon M, Fernandois D, Sauve F, Caron E, da Silva Lima N, Heras V, Martinez-Corral I, Mueller-Fielitz H (2021). Tanycytic networks mediate energy balance by feeding lactate to glucose-insensitive POMC neurons. J Clin Invest.

[58] Pellegrini L, Albecka A, Mallery DL, Kellner MJ, Paul D, Carter AP, James LC, Lancaster MA (2020). SARS-CoV-2 infects the brain choroid plexus and disrupts the blood-CSF barrier in human brain organoids. Cell Stem Cell.

[59] Chen R, Wang K, Yu J, Howard D, French L, Chen Z, Wen C, Xu Z (2020). The spatial and cell-type distribution of SARS-CoV-2 receptor ACE2 in the human and mouse brains. Front Neurol.

[60] Brann DH, Tsukahara T, Weinreb C, Lipovsek M, Van den Berge K, Gong B, Chance R, Macaulay IC, Chou HJ, Fletcher RB (2020). Non-neuronal expression of SARS-CoV-2 entry genes in the olfactory system suggests mechanisms underlying COVID-19-associated anosmia. Sci Adv..

[61] Meinhardt J, Radke J, Dittmayer C, Franz J, Thomas C, Mothes R, Laue M, Schneider J, Brunink S, Greuel S (2021). Olfactory transmucosal SARS-CoV-2 invasion as a port of central nervous system entry in individuals with COVID-19. Nat Neurosci.

[62] Onabajo OO, Banday AR, Stanifer ML, Yan W, Obajemu A, Santer DM, Florez-Vargas O, Piontkivska H, Vargas JM, Ring TJ (2020). Interferons and viruses induce a novel truncated ACE2 isoform and not the full-length SARS-CoV-2 receptor. Nat Genet.

[63] Blume C, Jackson CL, Spalluto CM, Legebeke J, Nazlamova L, Conforti F, Perotin JM, Frank M, Butler J, Crispin M (2021). A novel ACE2 isoform is expressed in human respiratory epithelia and is upregulated in response to interferons and RNA respiratory virus infection. Nat Genet.

[64] Sifat AE, Vaidya B, Kaisar MA, Cucullo L, Abbruscato TJ (2018). Nicotine and electronic cigarette (E-Cig) exposure decreases brain glucose utilization in ischemic stroke. J Neurochem.

[65] de Souza AR, Zago M, Eidelman DH, Hamid Q, Baglole CJ (2014). Aryl hydrocarbon receptor (AhR) attenuation of subchronic cigarette smoke-induced pulmonary neutrophilia is associated with retention of nuclear RelB and suppression of intercellular adhesion molecule-1 (ICAM-1). Toxicol Sci.

[66] Siddiqui R, Mungroo MR, Khan NA (1995). SARS-CoV-2 invasion of the central nervous: a brief review. Hosp Pract.

[67] Song E, Zhang C, Israelow B, Lu-Culligan A, Prado AV, Skriabine S, Lu P, Weizman OE, Liu F, Dai Y (2021). Neuroinvasion of SARS-CoV-2 in human and mouse brain. J Exp Med.

[68] Denton DA, McKinley MJ, Weisinger RS (1996). Hypothalamic integration of body fluid regulation. Proc Natl Acad Sci USA.

[69] Dantzer R (2018). Neuroimmune interactions: from the brain to the immune system and vice versa. Physiol Rev.

[70] Mussa BM, Srivastava A, Verberne AJM (2021). COVID-19 and neurological impairment: hypothalamic circuits and beyond. Viruses.

[71] Zhang BZ, Chu H, Han S, Shuai H, Deng J, Hu YF, Gong HR, Lee AC, Zou Z, Yau T (2020). SARS-CoV-2 infects human neural progenitor cells and brain organoids. Cell Res.

[72] Klempin F, Mosienko V, Matthes S, Villela DC, Todiras M, Penninger JM, Bader M, Santos RAS, Alenina N (2018). Depletion of angiotensin-converting enzyme 2 reduces brain serotonin and impairs the running-induced neurogenic response. Cell Mol Life Sci.

[73] Kanberg N, Ashton NJ, Andersson LM, Yilmaz A, Lindh M, Nilsson S, Price RW, Blennow K, Zetterberg H, Gisslen M (2020). Neurochemical evidence of astrocytic and neuronal injury commonly found in COVID-19. Neurology.

[74] Johanson C, PM CONN (2017). Choroid plexus-cerebrospinal fluid transport dynamics: support of brain health and a role in neurotherapeutics. Conn's translational neuroscience.

[75] Schurink B, Roos E, Radonic T, Barbe E, Bouman CSC, de Boer HH, de Bree GJ, Bulle EB, Aronica EM, Florquin S (2020). Viral presence and immunopathology in patients with lethal COVID-19: a prospective autopsy cohort study. Lancet Microbe.

[76] Neumann B, Schmidbauer ML, Dimitriadis K, Otto S, Knier B, Niesen WD, Hosp JA, Gunther A, Lindemann S, Nagy G (2020). Cerebrospinal fluid findings in COVID-19 patients with neurological symptoms. J Neurol Sci.

[77] Bhalerao A, Cucullo L (2020). Impact of tobacco smoke in HIV progression: a major risk factor for the development of NeuroAIDS and associated of CNS disorders. Z Gesundh Wiss.

[78] Tamayo-Velasco A, Martinez-Paz P, Penarrubia-Ponce MJ, de la Fuente I, Perez-Gonzalez S, Fernandez I, Duenas C, Gomez-Sanchez E, Lorenzo-Lopez M, Gomez-Pesquera E (2021). HGF, IL-1alpha, and IL-27 are robust biomarkers in early severity stratification of COVID-19 patients. J Clin Med.

[79] Zhang Z, Ai G, Chen L, Liu S, Gong C, Zhu X, Zhang C, Qin H, Hu J, Huang J (2021). Associations of immunological features with COVID-19 severity: a systematic review and meta-analysis. BMC Infect Dis.

[80] Lucas C, Wong P, Klein J, Castro TBR, Silva J, Sundaram M, Ellingson MK, Mao T, Oh JE, Israelow B (2020). Longitudinal analyses reveal immunological misfiring in severe COVID-19. Nature.

[81] Pons MJ, Ymana B, Mayanga-Herrera A, Saenz Y, Alvarez-Erviti L, Tapia-Rojas S, Gamarra R, Blanco AB, Moncunill G, Ugarte-Gil MF (2021). Cytokine profiles associated with worse prognosis in a hospitalized Peruvian COVID-19 cohort. Front Immunol.

[82] Yin SW, Zhou Z, Wang JL, Deng YF, Jing H, Qiu Y (2021). Viral loads, lymphocyte subsets and cytokines in asymptomatic, mildly and critical symptomatic patients with SARS-CoV-2 infection: a retrospective study. Virol J.

[83] Liu Y, Chen D, Hou J, Li H, Cao D, Guo M, Ling Y, Gao M, Zhou Y, Wan Y (2021). An inter-correlated cytokine network identified at the center of cytokine storm predicted COVID-19 prognosis. Cytokine.

[84] Gu T, Zhao S, Jin G, Song M, Zhi Y, Zhao R, Ma F, Zheng Y, Wang K, Liu H (2020). Cytokine signature induced by SARS-CoV-2 spike protein in a mouse model. Front Immunol.

[85] Cavalli G, Colafrancesco S, Emmi G, Imazio M, Lopalco G, Maggio MC, Sota J, Dinarello CA (2021). Interleukin 1alpha: a comprehensive review on the role of IL-1alpha in the pathogenesis and treatment of autoimmune and inflammatory diseases. Autoimmun Rev.

[86] Schon MP, Berking C, Biedermann T, Buhl T, Erpenbeck L, Eyerich K, Eyerich S, Ghoreschi K, Goebeler M, Ludwig RJ (2020). COVID-19 and immunological regulations - from basic and translational aspects to clinical implications. J Dtsch Dermatol Ges.

[87] Aldaham S, Foote JA, Chow HH, Hakim IA (2015). Smoking status effect on inflammatory markers in a randomized trial of current and former heavy smokers. Int J Inflam.

[88] Elisia I, Lam V, Cho B, Hay M, Li MY, Yeung M, Bu L, Jia W, Norton N, Lam S (2020). The effect of smoking on chronic inflammation, immune function and blood cell composition. Sci Rep.

[89] Khanna A, Guo M, Mehra M, Royal W (2013). Inflammation and oxidative stress induced by cigarette smoke in Lewis rat brains. J Neuroimmunol.

[90] Botelho FM, Bauer CM, Finch D, Nikota JK, Zavitz CC, Kelly A, Lambert KN, Piper S, Foster ML, Goldring JJ (2011). IL-1alpha/IL-1R1 expression in chronic obstructive pulmonary disease and mechanistic relevance to smoke-induced neutrophilia in mice. PLoS ONE.

[91] Paulson JR, Yang T, Selvaraj PK, Mdzinarishvili A, Van der Schyf CJ, Klein J, Bickel U, Abbruscato TJ (2010). Nicotine exacerbates brain edema during in vitro and in vivo focal ischemic conditions. J Pharmacol Exp Ther.

[92] Moynagh PN (2005). The interleukin-1 signalling pathway in astrocytes: a key contributor to inflammation in the brain. J Anat.

[93] Bailly L, Fabre R, Courjon J, Carles M, Dellamonica J, Pradier C (2022). Obesity, diabetes, hypertension and severe outcomes among inpatients with coronavirus disease 2019: a nationwide study. Clin Microbiol Infect.

